# T1-weighted Grey Matter Signal Intensity Alterations After Multiple Administrations of Gadobutrol in Patients with Multiple Sclerosis, Referenced to White Matter

**DOI:** 10.1038/s41598-018-35186-w

**Published:** 2018-11-15

**Authors:** Peter Kelemen, Jamila Alaoui, Dominik Sieron, Andrew Chan, Christian P. Kamm, Mirjam R. Heldner, Jan Gralla, Roland Wiest, Rajeev K. Verma

**Affiliations:** 1 University Institute of Diagnostic and Interventional Neuroradiology, Inselspital, University of Bern, Bern, Switzerland; 2 Institute of Radiology and Neuroradiology, Tiefenau Hospital, Inselgroup, Bern, Switzerland; 3 University Department of Neurology, Inselspital, University of Bern, Bern, Switzerland

## Abstract

The aim of the study was to investigate the signal-intensity-(SI)-ratio changes in the basal ganglia, the pulvinar thalami (PN), and the dentate nucleus (DN) using frontal white matter (FWM) as reference area, in patients with multiple sclerosis after frequent administrations of gadobutrol. A control group (group I) was compared to three stratified patient groups (group II: mean applications of gadobutrol 3.7; group III: 7.5 applications; group IV: 13.8 applications). SI-ratios of the pallidum, putamen, caudate nucleus, and pulvinar thalami were calculated with: 1. FWM, and 2. PN. DN-to-pons and DN-to-FWM ratios were also calculated. The most significant SI-ratio-changes were found by comparing group I and IV for both reference values. However, by using FWM as reference an SI-ratio increase was observed, while an SI-ratio decrease was seen if referenced to the PN. DN-to-FWM showed an SI-ratio increase, too. The PN revealed a significant SI-ratio increase itself, correlating with the number of gadolinium applications, when referenced to FWM. Therefore, SI-ratio calculations using the thalamus as reference might be flawed. In addition, a minor gadolinium accumulation is possible, if FWM was used as reference area. Further studies are necessary to verify our results.

## Introduction

Contrast-enhanced MRI is indispensable when diagnosing pathological tissue alterations. Gadolinium-based contrast agents (GBCA) have been used worldwide for more than two decades. Free gadolinium (Gd^3+^) is highly toxic^1^ and is therefore bound to a chelate molecule in contrast agents. These contrast agents can be divided into two binding forms: linear chelates with an open chain, and macrocyclic chelates with a non-open chain^2,3^. Studies have reported that linear structured GBCAs release more toxic Gd^3+^ than GBCAs with a macrocyclic structure^2,4^. Hence, macrocyclic GBCAs are considered more stable.

Recent studies have reported an increased signal intensity (SI) in the globus pallidus and dentate nucleus (DN) in patients that received repetitive GBCA administrations, especially if the GBCA had a linear structure^5,6^. These findings suggest an accumulation of gadolinium (Gd) in these areas. A deposit of Gd in cadaveric brain tissue of patients after multiple GBCA administrations also was recently reported^7–9^.

In most studies that investigated the macrocyclic GBCA gadobutrol no SI-ratio changes were detected in the globus pallidus on T1w images after repetitive administrations. Only Stojanov *et al*. reported an SI increase in both the globus pallidus and DN after multiple administrations of gadobutrol^10^, whereas other studies reported conflicting results without SI increase in globus pallidus and/or DN after multiple intravenous injections of gadobutrol^11,12^.

In these studies^10–12^ the thalamus was used as the reference area to determine the signal intensity (SI) changes of the globus pallidum. However, McDonald *et al*. found Gd in postmortem analyses using inductively coupled plasma mass spectroscopy (ICP-MS). In addition, a significant SI increase in the pulvinar thalami in T1w images was found, correlating with the cumulative intravenous GBCA dose (mL). If the thalamic tissue accumulates Gd itself, the SI increase in T1-weighted images might be underestimated if thalamus is used as a reference area. In contrast, for white matter distinctly lower Gd deposits were reported in micrograms/gram tissue after multiple GBCA administrations compared to the basal ganglia^7–9^. Therefore, the WM might be a more reliable reference value for the evaluation of signal changes in the globus pallidus, in other basal ganglia, and the thalamus itself. Given this hypothesis, the aim of this study was to evaluate a potential correlation between the number of gadobutrol administrations and the SI in precontrast T1-weighted images in the basal ganglia (globus pallidus, head of the caudate nucleus, putamen), and the pulvinar of the thalamus by using the frontal WM as the reference value. As an SI increase had been reported for the pulvinar thalami, we selected this area of the thalamus for investigation^7^. For comparison with the previous studies, a further aim was to use the pulvinar thalami as reference value for ratio calculations. We also calculated DN-to-pons ratio differences for additional comparisons. To evaluate the pons as reference area, we additionally calculated the DN-to-frontal WM ratio and the frontal WM-to-pons ratio.

## Materials and Methods

### Patient data

The retrospective study was approved by the local Ethics Committee (KEK BE, Antrag Nr. 356/15; Kantonale Ethikkommission Bern, Switzerland) and the methods were carried out in accordance with the relevant guidelines and regulations. The informed consent of the patients was not necessary due to the retrospective study design.

### Patients and Healthy Volunteers

Data of the patients with MS or clinically isolated syndrome (CIS) who underwent MRI with intravenous application of gadobutrol at our institute between April 2011 and May 2015 was identified. The patient’s inclusion criteria were: a diagnosis of MS or CIS (according to the 2010 McDonald’s criteria^13^), and at least one previous intravenous gadobutrol administration (Gd-BT-DO3A at a dosage of 0.1 mL/kg body weight; Gadovist, Bayer Healthcare, Berlin, Germany). Gadobutrol has been exclusively administered in our Institute since 2002. Patients were excluded if they had received any GBCA at all during their lifetime, from their recollection, before receiving Gadobutrol in our department. Furthermore, if possible, the first symptom onset should have been during or after 2002, and the patient´s work-up should have been performed at our center, including the MR examination follow-ups. If the first symptom onset was before 2002, patients should have had a long-term treatment of at least six years in our center.

The inclusion criteria for the healthy control group was no administration of GBCA during their lifetime at all. For all subjects, acquisition of standardized T1-weighted images with identical parameters in the identical 3 T MRI was mandatory. Renal insufficiency, poor imaging quality (e.g. due to motion artifacts), and other CNS diseases were ruled out.

The control group was defined as group I. The patients were divided into three groups, depending on the number of gadobutrol administrations: group II (a total of 1 to 5 administrations), group III (6 to 10), and group IV (>10).

Inclusion criteria for all subjects were that all images were acquired in the same MRI scanner (Siemens Magnetom Verio 3 T) and an identical protocol.

### MR Imaging Acquisition and Protocol

T1-weighted images (3D MPRAGE) of all subjects were acquired with identical parameters: voxel size 1.0 × 1.0 × 1.0 mm, TR = 2530 ms, TE = 2.96 ms, TI = 1100 ms, flip angle 7°, FoV read 250 mm, acquisition time 4:30 min.

### Image Analysis

**Figure 1:**
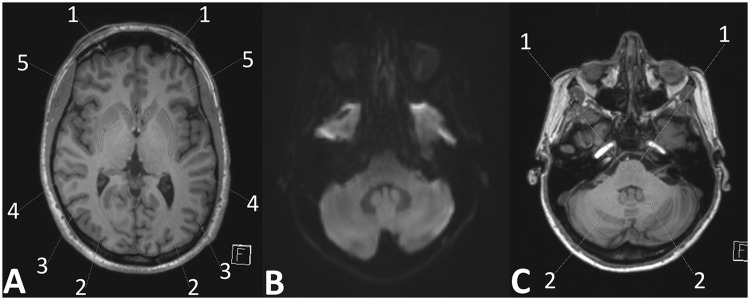
Image analysis/ROI measurements of the basal ganglia, thalamus, and the frontal white matter. Image analysis/ROI measurements of the DN and pons. (**A**) T1-weighted image of a healthy 39-year-old male volunteer. As shown, all ROI measurements of signal intensities were made in this standardized manner; Selection of ROIs was as follows: 1 Frontal WM: The chosen ROI was in a standardized and identical frontal WM location and attention was paid to the homogeneity of the area. 2 Thalamus: The thalamus already shows unequal and different SI in itself on unenhanced T1-weighted images of healthy subjects (slightly more hyperintense in the anterolateral parts, and hypointense in the dorsomedial/pulvinar-adjacent parts). Also, as described and demonstrated in^7^ the pulvinar of the thalamus shows SI increase after multiple GBCA administrations. We chose the pulvinar thalami as the ROI for two reasons: a) it is a relatively signal-homogeneous area on T1-weighted images compared to other parts of the thalamus and therefore convenient to identify visually for drawings, and b) according to^7^ if any SI increase after GBCA administrations can be expected at all, the pulvinar thalami is the most likely area. 3 Globus pallidus shows a relatively high signal on T1-weighted images and appears partly with a fluid transition into the internal capsule. The most easily identifiable area (ROI) is in the rostral part of the globus at the base of its trigonal shape. 4 and 5. Putamen and head of the caudate nucleus: both basal ganglia are homogeneously hypointense on T1-weighted images and well delineated. (**B**) Diffusion weighted image (DWI) and 1C) T1-weighted image of a 47-year-old female MS patient. To clearly identify the DN the DWI was used. ROI delineations were copied to the T1-weighted image for SI-measurements in a standardized manner; 1 = pons (white matter), 2 = DN.

Basal ganglia, DN measurements, frontal WM and pons were each made on a sole transversal T1-weighted image slice (Fig. 1). Since we expected no or at best minimally significant SI changes in the T1-weighted images, we defined the anatomical structures of the basal ganglia and thalamus in the T1-weighted images in a standardized manner for the SI-ratio calculations: The region of interest (ROI) was drawn on the native T1-weighted images. All drawings were made in a standardized manner. ROI positions were checked by two diagnostic neuroradiologists (13 and 8 years of experience in radiology) and, if necessary, corrected to assure an identical anatomical location. Subsequent analysis was performed and both radiologists were blinded to the number of Gd administrations. All areas were analyzed bilaterally. ROIs were selected on the T1-weighted images in anatomically homogeneous, and thus, easily reproducible areas. For detailed information see Fig. 1.

### Statistical analysis

Analyses were conducted using SPSS Statistics 24 (IBM, Armonk, NY). To normalize measured SI values, three classifications of SI-ratios were calculated:

1. The SI values of all basal ganglia and the thalamus (pulvinar) were divided by the SI value of the frontal WM (reference region).
2. The SI values of all basal ganglia were divided by the SI value of the pulvinar thalami (reference region).
3. The SI values of the dentate nucleus were divided by the SI values of the pons and the frontal white matter (reference regions). SI values of the frontal white matter was divided by the SI values of the pons (reference region).

Levene’s test was applied to confirm the equality of variances for the SI-ratio values for both classifications and all four groups, followed by an analysis of variance (ANOVA) for assessment of the SI-ratio differences to test whether or not the SI-ratio means of the four groups were equal. For both classifications, group I was compared to groups II, III, and IV individually and to groups II–IV together. Furthermore, to estimate the potential influences of the demyelinating disease itself, the MS group with lowest gadobutrol administrations (group II) was compared to the MS group with the highest (group IV). A p-value below 0.05 was considered to be significant.

## Results

### Study Cohort (Healthy Volunteers and Patients)

Twenty healthy volunteers (group I), no prior GBCA administration at all; 11f; mean age 37.1y) and a total of 77 patients with MS or CIS (57f; mean age 44.5y) met the inclusion criteria (4 with CIS, 61 with relapsing-remitting MS, 11 with secondary progressive multiple sclerosis, and one with primary progressive multiple sclerosis). Furthermore, 20 patients were assigned to group II, 35 to group III, and 22 to group IV. Of the 77 patients, 66 (85.7%) had their first symptom onset after 2002 and the complete work-ups, including all follow-up MR examinations, were performed in our MS center (group II with 75%, group III with 88.6%, and group IV with 90.9%), while the remaining 11 had a long-term treatment of at least six years in our center with all follow-up MRI examinations. For demographic and group characteristics see Table 1.

**Table 1:** Demographic Characteristics of the Study Cohort.

Parameter	Group I (healthy probands)	Group II (1–5 Gadobutrol administr.)	Group III (6–10 Gadobutrol administr.)	Group IV (>10 Gadobutrol administr.)	Group II–IV (all MS patients)
No. of subjects	20	20	35	22	77
Mean age (y)	37.1	46.4	47.8	37.6	44.5
Sex (f/m)	11/9	13/7	26/9	18/4	57/20
Mean no. of MRI examinations with gadobutrol (stdev.)	0	3.65 (1.04)	7.51 (1.29)	13.83 (3.04)	8.3 (4.26)
Mean interval between gadobutrol administrations in days (stdev)	0	553.0 (288.93)	454.26 (314.22)	300.14 (209.89)	435.86 (293.78)
Mean EDSS (stdev)	—	3.13 (1.96)	2.7 (1.6)	2.61 (1.47)	2.75 (1.66)
Mean illness duration (stdev)	—	12.46 (11.88)	8.4 (6.09)	9.8 (5.37)	9.83 (7.91)

Legend: EDSS = Expanded Disability Status Scale; MS = Multiple Sclerosis; stdev = Standard deviation.

### Visual detectability of SI increase in the basal ganglia, the thalamus and DN

No clear SI increase in the basal ganglia, the thalamus and DN was visually detectable in the patient groups and the control group.

### SI-ratio Results of Basal Ganglia, the Pulvinar Thalami, and Dentate Nucleus referenced by White Matter

**Figure 2:**
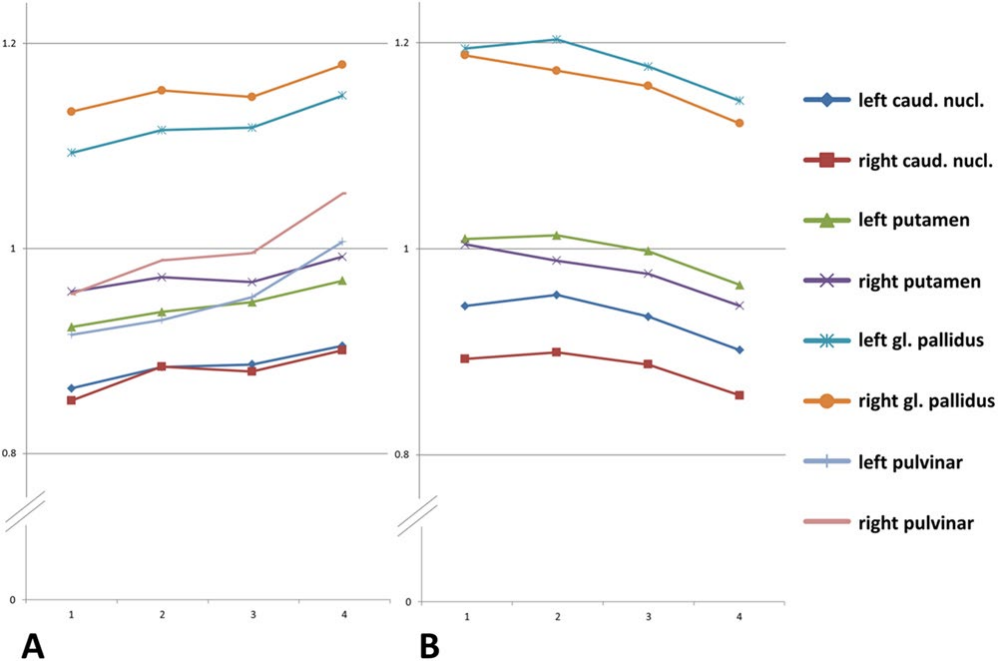
Mean of SI-ratio using frontal WM as reference (**A**) and the pulvinar thalami as reference (**B**). Mean of the SI-ratio for each group (Y-axis: SI-ratio values; X-axis: 1 = group I, 2 = group II, 3 = group III, 4 = group IV.). As demonstrated in (**A**) by using the white matter as reference, SI-ratio of the basal ganglia and the pulvinar thalami increases from group I to group IV (p-values between <0.001 and 0.032 for comparisons between group I and IV). If the pulvinar thalami is used as reference (**B**) the SI-ratio of the basal ganglia decreases (p-values between <0.007 and 0.052 for comparisons between group I and IV). For individual standard deviations and standard errors see Supplementary Tables S1 and S2.

Significant increases in the SI-ratios between group I (control group) and all patients with MS or CIS (groups II–IV together) were found in 6 of 8 areas (p-values between 0.001 and 0.04). Comparison of group I and IV revealed a significant increase in all areas (8 of 8; p-values between <0.001 and 0.29), while only one significant increase was found comparing group I with group II, and two increases if compared to group III. See Fig. 2A for illustration and Supplementary Table S1 for details (supplementary information).

Additionally, we evaluated the dentate nucleus where a significant increase was only found between group I and group IV on both sides (p = 0.04 and 0.002; last two rows in Supplementary Table S1).

### SI-ratio Results for Basal Ganglia Referenced by the Pulvinar Thalami

Significant decrease in the SI-ratios between group I and all patients with MS or CIS (groups II–IV) was found in only 1 of 6 areas (right putamen; p = 0.038). No significant differences were found in comparison to group II or group III. The comparison to group IV, however, revealed a significant decrease in the SI-ratios in five of six regions (p-values between 0.007 and 0.04). See Fig. 2B for illustration and Supplementary Table S2 for details.

Noteworthy concerning the reference areas: an increase of the SI-ratio was seen if the frontal WM was used, while a decrease in the SI-ratio was found if the pulvinar thalami was used as the reference.

### SI ratio Differences Between Group II and Group IV Referenced by the Frontal WM and the Pulvinar Thalami

The non-lesional white matter in MS is called normal-appearing white matter (NAWM). SI values may have been impacted because of an endogeneous inflammatory reaction throughout the whole white matter, therefore, a comparison of MS groups with the lowest and highest gadobutrol administration was additionally performed (group II vs. IV).

Referenced by the WM, a significant SI-ratio increase was found in three supratentorial areas (left putamen, left and right pulvinar thalami; p-values between <0.001 and 0.027). Referenced by the pulvinar thalami, a significant SI-ratio decrease was found in all areas (6 of 6; p-values between 0.005 and 0.036). For details see Supplementary Table S3.

Additionally, a significant bilateral SI-ratio increase was found in the infratentorial dentate nucleus, if referenced to the frontal WM (p-values 0.003 and 0.005; see last two rows of Supplementary Table S3).

Again, if the SI values were referenced to the frontal WM a significant SI increase was observed (in 3 supratentorial areas and in the dentate nucleus), while an SI decrease was detected if referenced to the pulvinar thalami.

### SI-ratio results of DN-to-Pons and Frontal WM-to-Pons SI-Ratio Comparisons

**Figure 3:**
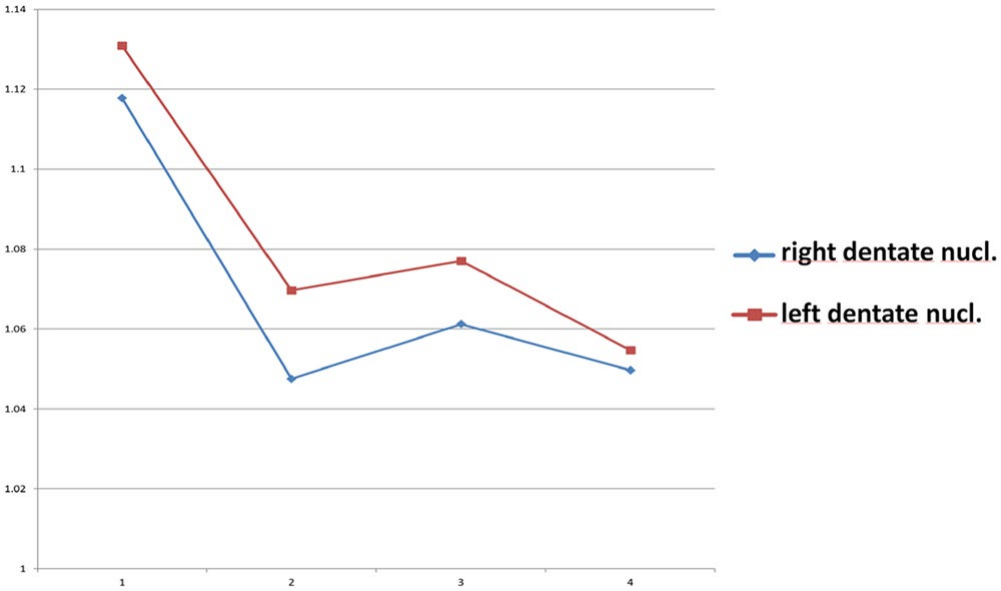
Mean of DN-To-pons- SI-ratio. Mean of the SI-ratio for each group (Y-axis: SI-ratio values; X-axis: 1 = group I, 2 = group II, 3 = group III, 4 = group IV.). As mentioned, the control group showed the highest ratio values, whereas the MS groups revealed comparable values (p-values: 0.009 and 0.01, respectively for comparisons between group I and IV). For individual standard deviations and standard errors see Supplementary Table S4.

For comparisons with other studies, DN-to-pons SI-ratio calculations were performed in the same manner as the basal ganglia calculations. Comparing group I with groups II, III, IV, and All Patient Groups (II–IV) significant decreases in seven of eight calculations can be seen (p-values between 0.008 and 0.037). Of note, a significantly higher SI value was found in the control group that had received no GBCA in their lifetime. No significant difference was found among MS groups II and IV. See Supplementary Table S4 for details (supplementary information) and Fig. 3 for illustration.

The frontal WM-to-pons ratio revealed a significant bilateral SI-ratio decrease (p-values between 0.001 and 0.029). Further, a significant decrease was observed among MS groups II and IV (p-values 0.01 and 0.027); for details see last two rows of Supplementary Table S4.

## Discussion

Our descriptive study revealed some discrepant findings regarding the T1w SI-ratio changes in the basal ganglia and the thalamus, in contrast to the DN.

First, by using the frontal WM as reference value, we found a significant increase in SI-ratio between the control group (group I) and all patients in six of the eight supratentorial regions studied, while only one of six regions showed a significant SI-ratio decrease if the pulvinar thalami was used as reference.

Furthermore, comparing group I and group IV (mean of 13.83 gadobutrol administrations; mean interval between administrations 300.1 days) with frontal WM as reference, a significant increase in the SI-ratio was found in all areas measured, but only in two areas when compared with group III (7.51 administrations; 454.3 days), and only in one area when compared to group II (3.65 administrations; 553.0 days). The largest SI-ratio increase was seen in the pulvinar thalami. Hence, as illustrated in Fig. 2 and Supplementary Table S2 (last column) the mean SI-ratio decreases if the pulvinar thalami is used as the reference value.

Several investigations of gadobutrol used the thalamus for region-based analysis^5,6,10–12^. McDonald *et al*.^7^ used the SI of the cerebrospinal fluid as the reference value and revealed an SI increase in the pulvinar of the thalamus on native T1-weighted images in their investigation of gadodiamide, a linear non-ionic GBCA. However, comparing the illustrated ROIs of the thalamus in the various studies (e.g.)^5,7,10,12^ shows that no consistent, standardized measurement was made (some authors referred to the methodology of Kanda *et al*. without an illustration^6,11^). A standardized measurement of the thalamus is mandatory for two reasons. First, the thalamus itself shows varying SI in T1-weighted images which can already be both visually detected and quantitatively measured in healthy probands. While higher SI is found in the anterolateral parts of the thalamus, the area of the pulvinar thalami shows lower SI. Second, as illustrated in^7^, the pulvinar thalami region already shows visual SI increases after gadodiamide administrations, making the thalamus a potentially flawed reference area for normalization. Correlating to this, Marie *et al*. very recently revealed a significant SI-ratio increase of the pulvinar in T1w images referenced to the WM after at least 10 consecutive doses of gadodiamide, a linear GBCA^14^.

Our results from comparing group I with group II and group III (mean 3.65 and 7.51 gadobutrol administrations), using the pulvinar thalami as reference value, could correlate with several previous studies^6,11,12,15^ where no significant SI changes of the globus pallidus were reported (mean 7.3, ≥5, 1, and ≥3 gadobutrol administrations, respectively). One very recent study has revealed similar results with a significant decrease in the GP/Th SI-ratio associated with the number of macrocyclic ionic GBCA inter alia and, therefore, an SI increase in the thalamus^16^. Looking closer to the illustrated measurement of the thalamus in that investigation it is quite comparable to our measurements including parts of the pulvinar thalami. Another very recent investigation revealed a significant T1 shortening in the globus pallidus in 46 subjects with a mean of nine gadobutrol administrations, but no GP/Th SI-ratio increase^17^. Again, this discrepant finding might be explained by the thalamus as reference area. In contrast Müller *et al*. found no changes in relaxation time in 17 subjects with a mean of 8 gadobutrol administrations, even by using the frontal white matter for normalization^18^. Although the number of adminstrations is comparable to Kang’s study and our study, the number of patients is distinctly lower, which might explain the different results. Bhargava *et al*. found no SI-ratio increase in the globus pallidus referenced to the corpus callosum in children with brain tumors after having received either five or more gadobutrol adminstrations (46 patients) or more than 14 administrations (6 patients)^19^. Despite that no significant increase was found in the first group, the results are comparable to our group II (bilaterally no SI-ratio increase with mean gadobutrol administrations of 7.51; pt. no. 20) and all MS patients with a higher patient number (groups II-IV; mean gadobutrol administrations 8.3, pt. no. 77) with an SI-ratio increase only on the left (p = 0.033), but not on the right (p = 0.119). As mentioned by the authors, a sampling error may apply to the second group with a small sample size of only six patients, which might explain the different finding compared to our group IV.

However, as an unexpected and coincidental new finding, significant decreases between the healthy control group and the MS groups were revealed in seven of eight calculations when calculating the DN-to-pons-ratio (p-values between 0.008 and 0.037; Supplementary Table S4). Presumed previous administrations of linear GBCA, radiation therapy, or chemotherapy certainly can be ruled out in the healthy control group. An age related T1w signal increase can be further ruled out, since group I was the youngest of all groups. In accordance with most other studies (with comparable gadobutrol administrations) no DN-to-pons SI-ratio increase between MS-patient groups II and IV was found (lowest vs. highest gadobutrol administrations; p = 0.561 and 0.932). However, a decrease in DN-to-pons ratio has been noted in several studies after multiple administrations of macrocyclic GBCA in patients with MS and other diseases^20–22^. Reasons for the SI decrease were discussed in these investigations, such as the “wash out hypothesis” over time, the dechelation hypothesis, excess ligand in the formulation of gadobutrol, or an SI increase in the pons itself. In our study we went one step further by integrating the frontal WM for ratio calculations. This revealed some surprising findings: a significant DN-to-frontal WM SI-ratio increase and a significant frontal WM-to-pons decrease between the control group and group IV (Supplementary Tables S1 and S4). The findings imply a higher SI-ratio increase in the pons, compared to the frontal WM. Therefore, a potentially higher gadolinium deposit or delayed “wash out effect” in the pons might be an explanation for our results. A dechelation of gadolinium is unlikely, because the T1w hyperintense signal effect would disappear, but in the pons a T1w increase was observed in relation to the frontal WM. The presence of excess ligand can either not explain the SI-ratio increase of the pons. “Free” ligand is distributed all over the body tissue and would have a comparable affinity to gadolinium e.g. in the frontal WM, therefore no significant differences in the frontal WM-to-pons-ratio would be expected. Besides the GBCA application other factors could influence T1w signal alterations. A reason might be the disease of the patients. As the DN-to-pons SI value is comparable among the MS groups, but significantly lower compared to the control group, it cannot be ruled out that the illness or illness therapy causes SI decreases in the DN, e.g. a correlation of T2w hyper- or hypointense DN changes has been previously described depending on the immunomodulatory therapy in MS^23^. A reduction of brain iron due to immunomodulatory medication was discussed.

Age, illness severity, and disease duration correlate with SI changes of the deep gray matter in MS patients, especially with T2w susceptibility alterations, that might influence T1 signal contrarivise^24^. A comparison of the groups II and IV (lowest vs. highest gadolinium administration) revealed increased SI values in group IV, if referenced to the WM, and decreased SI values, if referenced to the pulvinar (Supplementary Table S3). These findings stand in opposition to the expected changes caused by MS: age, illness severity (EDSS) and disease duration were the highest in group II, while age and illness severity were the lowest in group IV (Table 1). From this point of view, the SI changes in the basal ganglia and the thalamus in our study cannot be explained by MS.

### Comparisons with Non-ratio Investigations

In a non-ratio, voxel-based whole-brain analysis, Langner *et al*. found no significant changes in SI of gray matter and WM after up to five mcGBCA gadobutrol administrations (mean 2.2)^25^. These results are in line with our results if comparing group I with group II (low number of gadobutrol administration), only one area, and with group III only two areas of significant difference were revealed (mean administration no. 3.7 and 7.5 respectively) (Supplementary Table S1).

Regarding non-MRI studies, our findings might correlate with the afore-mentioned investigations of human autopsy brain specimens^7–9^, where accumulations in the basal ganglia, as well as in the thalamus were found after administrations of linear and macrocyclic GBCA.

Some recently published preclinical investigations examined rat brain tissue after multiple administrations of linear and macrocyclic GBCA using ICP-MS^26–28^. Lohrke *et al*. examined differences between linear and macrocyclic GBCA in brain tissue of rats histopathologically^28^ and reported that the average detected residual Gd concentration in brain tissue was approximately 15 times higher for linear than for macrocyclic GBCAs. McDonald *et al*. reported a neuronal tissue deposit of gadolinium in both macrocyclic and linear GBCAs, albeit at lower concentrations than with macrocyclic agents^26^. This might lend support to our findings that a high average number of gadobutrol administrations (13.83; group IV) was necessary for revealing significant differences between SI-ratios in all areas analyzed. Even then the changes were not clearly visible in patients, that had received gadobutrol up to 28 times and SI-ratio changes were in the hundredths range. This is in contrast to MRI studies on linear GBCA, e.g. Kanda *et al*., that stated T1w hyperintensity appeared with more than five past adminsitrations of linear GBCA^29^.

Frenzel *et al*. found comparable amounts of Gd after three and 24 days in the soluble brain fraction in rats treated with a very high dosage of linear versus macrocyclic GBCA, including gadobutrol^27^. The amount of Gd in the insoluble fraction of the brain tissue was much higher in rats treated with linear GBCA (Gd-DTPA, gadobenate, and gadodiamide) than in rats treated with gadobutrol. The study indicates a “washout” effect of macrocyclic GBCA in brain tissue. While linear GBCAs seem to release Gd into the brain tissue, macrocyclic GBCAs retain their molecule structure and therefore have the potential to be eliminated over time. In our study the most significant rise in the SI-ratio correlated with the number of gadobutrol administrations (group IV), but also with the time interval between administrations. The shortest time interval between MRI examinations was found in group IV (300.1 days) with the highest number of significant differences, while the longest interval was found in group II (553.0 days) with the lowest number of significant differences. Therefore, a “washout” effect must be considered, which might be more advanced and pronounced in group II than in group IV. Neither can one rule out the possibility that the SI-ratio might even decrease through a “washout” effect over time in group IV if no additional GBCA is given. Our study stratified groups according to the number of gadolinium administrations. However, for future investigations, a stratification of groups according to the time interval between administrations of macrocyclic GBCA would be very helpful to assess the extent of a “wash out” effect of soluble gadolinium proportions.

### Strengths and Limitations

One strength of this study is that all subjects (patients and volunteers) underwent an MRI in the same 3 T MR scanner with the same protocol including identical parameters. T1-weighted MPRAGE was applied, which is recommended for research purposes, because it correlates better with quantitative analysis offering advantages over SE sequences^30^. Only macrocyclic gadobutrol was administered to the study cohort of MS and CIS patients. Moreover, the reference group (group I) for group comparisons had no intravenous GBCA at all in their lifetimes. And finally, equally signal-intense frontal WM was used as a reference for SI-ratio calculations.

The study has several limitations. The number of study subjects in each group is relatively small and the comparison of group I with group IV, where the most significant changes were found, had only 20 versus 22 subjects. Although Gd accumulation in frontal WM is low, it might lead to an underestimation of the SI increase because of a potential minimal SI increase in the WM itself. Only patients with MS and CIS were examined. Although typical causes of SI alterations in the grey matter due to MS are very unlikely, an influence of SI on both the grey and white matter due to MS/CIS cannot be entirely ruled out (an influence of immunomodulatory therapy in particular). Additionally, although MS groups II and IV were compared with each other, NAWM inflammation is not visible to MRI and might cause different SI values in these groups. All drawings of ROIs were made manually and therefore prone to bias, but were made in a standardized manner. This investigation analyzed patients inter-individually. An intra-individual approach, examining patients over the course of time after gadobutrol administrations would be more accurate. Furthermore, SI changes were only detected by intensity values, not visually, suggesting at most a minimal Gd accumulation. Lastly, a complete exclusion of previous linear GBCA administration cannot be fully guaranteed in any retrospective study. However, we think this is unlikely since this would have been most likely for group II with its longest illness duration (mean 12.46y), highest EDSS (mean 3.13) and the lowest number of patients (75%) with an exclusive work-up in our center.

Notwithstanding that no significant SI increase in DN-to-pons ratio was found after multiple administrations of gadobutrol in accordance to other studies, our study demonstrates elevated SI-ratios in the globus pallidus, the putamen, the head of the caudate nucleus, the dentate nucleus and the pulvinar thalami in reference to frontal WM. The SI increases occurred particularly in patients who had received a high number of gadobutrol administrations (mean 13.83) and a short time interval in between. Hence, an accumulation and/or a delayed “wash out” of gadolinium cannot be ruled out in these grey matter areas and DN-to-pons SI-ratio should be interpreted and evaluated separately. Additionally, globus pallidus-to-thalamus ratio calculations might be flawed and an uncertain measurement method for evaluating T1w SI increases due to gadolinium accumulation. However, in contrast to previous studies with linear GBCA, the changes were obscured by visual analysis. These findings suggest, that despite a higher average number of GBCA administrations, Gd accumulation in brain tissue is far lower for macrocyclic gadobutrol than for linear GBCAs. Further studies with a larger cohort, a high number of gadobutrol administrations, and intra-individual comparisons of SI changes are needed to verify our findings.

## Electronic supplementary material

Supplementary Information

## Data Availability

The datasets generated and/or analyzed during the current study are not publicly available due to preclusion from dissemination according to Swiss federal law regulations but are available from the corresponding author on reasonable request.
