# Supplementary material for: T1-weighted Grey Matter Signal Intensity Alterations After Multiple Administrations of Gadobutrol in Patients with Multiple Sclerosis, Referenced to White Matter
Source: Sci Rep. 2018 Nov 15;8:16844. doi: 10.1038/s41598-018-35186-w (PMC6237839; doi:10.1038/s41598-018-35186-w)
Supplement: Supplementary file 1 — Supplementary Information [file 41598_2018_35186_MOESM1_ESM.docx]

**Supplementary information**

**Title: T1-weighted Grey Matter Signal Intensity Alterations After Multiple Administrations of Gadobutrol in Patients with Multiple Sclerosis, Referenced to White Matter**

Peter Kelemen (MD)^1^, Jamila Alaoui (MD)^1^, Dominik Sieron (MD)^2^, Chan Andrew (MD)^3^, Christian P Kamm (MD)^3^, Mirjam R Heldner (MD)^3^, Jan Gralla (MD)^1^, Roland Wiest (MD)^1^, Rajeev K Verma (MD)^1,2*^

^1^University Institute of Diagnostic and Interventional Neuroradiology, Inselspital, University of Bern, Switzerland

^2^Institute of Radiology and Neuroradiology, Tiefenau Hospital, Inselgroup, Bern, Switzerland

^3^University Department of Neurology, Inselspital, University of Bern, Switzerland

Corresponding address:

Rajeev Kumar Verma (MD)

Institute of Diagnostic and Interventional Neuroradiology

Inselspital, University Hospital Bern

Freiburgstrasse 4

3010 Bern, Switzerland

Email: [verma@gmx.ch](mailto:verma@gmx.ch)

**Supplementary Table S1**

**SI-Ratio Comparisons of Group I with Group II, III, IV and all Patient Groups (II–IV) with Frontal WM as the Reference Value**

|  | **Group I** | **Group II** | **Group III** | **Group IV** | **Groups II-IV (all)** | **SI difference Group I vs. Group IV** |
| --- | --- | --- | --- | --- | --- | --- |
| Left caudate nucleus | 0.86375/ 0.04796/ 0.01072/ 0.8413 - 0.8862 | 0.88458/ 0.048801/ 0.010912/ 0.86174 - 0.90741/ (0.142) | 0.88704/ 0.034160/ .005774/ 0.87531 - 0.89878/ (0.065) | 0.90524/ 0.051296/ 0.010936/ 0.88250 - 0.92799/ **(0.003*** | 0.88586/ 0.045872/ 0.004658/ 0.87661 - 0.89510/ **(0.013*)** | +0.04149 |
| Right caudate nucleus | 0.85165/ 0.04415/ 0.00987/ 0.83099 - 0.87231 | 0.88478/ 0.036872/ 0.008245/ 0.86752 - 0.90203/ **(0.017*)** | 0.87993/ 0.035945 /0.006076/ 0.86758 - 0.89228/ **(0.021*)** | 0.90077/ 0.055898/ 0.011917/ 0.87598 - 0.92555/ **(<0.001*)** | 0.87982/ 0.045464/ 0.004616/ 0.87066 - 0.88899/ **(0.001*)** | +0.04912 |
| Left putamen | 0.92355/ 0.048194/0.010777/ 0.90099 - 0.94611 | 0.93818/ 0.041558/ 0.009293/ 0.91873 - 0.95762/ (0.296) | 0.94776/ 0.037056/ 0.006264/ 0.93503 - 0.96049/ (0.053) | 0.96874/ 0.051869/ 0.011059/ 0.94574 - 0.99174/ **(0.001*)** | 0.94555/ 0.045974/ 0.004668/ 0.93628 - 0.95482/ **(0.013*)** | +0.04519 |
| Right putamen | 0.9579/ 0.049393/ 0.011045/ 0.93478 - 0.98102 | 0.9721/ 0.036163/ 0.008086/ 0.95518 - 0.98902/ (0.37) | 0.96737/ 0.047005/ 0.007945/ 0.95122 - 0.98352/ (0.5) | 0.992/ 0.063563/ 0.013552/ 0.96382 - 1.02018/ **(0.029*)** | 0.97198/ 0.050487/ 0.005126/ 0.96180 - 0.98215/ (0.13) | +0.0341 |
| Left globus Pallidus | 1.0935/ 0.062351/ 0.013942/ 1.06432 - 1.12268 | 1.11558/ 0.058245/ 0.013024/ 1.08832 - 1.14283/ (0.266) | 1.11783/ 0.059094/ 0.009989/ 1.09753 - 1.13813/ (0.168) | 1.14922/ 0.070869/ 0.015109/ 1.11780 - 1.18064/ **(0.005*)** | 1.11947/ 0.064217/ 0.006520/ 1.10652 - 1.13241/ **(0.033*)** | +0.05572 |
| Right globus pallidus | 1.1334/ 0.058328/ 0.013043/ 1.10610 - 1.16070/ 1.041 | 1.15418/ 0.045931/ 0.010270/ 1.13268 - 1.17567/ (0.335) | 1.1479/ 0.074684/ 0.012624/ 1.12225 - 1.17355/ (0.448) | 1.17902/ 0.079681/ 0.016988/ 1.14369 - 1.21434/ **(0.032*)** | 1.15326/ 0.068556/ 0.006961/ 1.13944 1.16708 (0.119) | +0.04562 |
| Left pulvinar thalamus | 0.91615/ 0.054204/ 0.012120/ 0.89078 - 0.94152 | 0.93038/ 0.061001/ 0.013640/ 0.90183 - 0.95892/ (0.472) | 0.95259/ 0.060540/ 0.010233/ 0.93179 - 0.97338/ **(0.04*)** | 1.00648/ 0.072199/ 0.015393/ 0.97447 - 1.03850/ **(<0.001*)** | 0.95272/ 0.069292/ 0.007036/ 0.93875 - 0.96668/ **(0.04*)** | +0.09033 |
| Right pulvinar thalamus | 0.95575/ 0.057888/ 0.012944/ 0.92866 - 0.98284 | 0.98868/ 0.059586 0.013324 0.96079 1.01656 (0.151) | 0.99579/ 0.077019/ 0.013019/ 0.96933 - 1.02224/ (0.05) | 1.05356/ 0.083831 0.017873 1.01639 1.09073 **(<0.001*)** | 0.99917/ 0.078115/ 0.007931/ 0.98342 - 1.01491/ **(0.002*)** | +0.09781 |
| Left dentate nucleus | 1.0418/ 0.07269/ 0.01625/ 1.0078 - 1.0758 | 1.0166/ 0.10254/ 0.02293/ 0.9686 - 1.0646/ (0.359) | 1.0398/ 0.08426/ 0.01424/ 1.0108 - 1.0687/ (0.934) | 1.1210/ 0.08438/ 0.01799/ 1.0836 - 1.1584/ **(0.04*)** | 1.0538/ 0.09286/ 0.00943/ 1.0351 - 1.0725/ (0.427) | +0.0792 |
| Right dentate nucleus | 1.0885/ 0.08578/ 0.01918/ 1.0484 - 1.1287 | 1.0916/ 0.09262/ 0.02071/ 1.0482 - 1.1349/ (0.917) | 1.0944/ 0.09233/ 0.01561/ 1.0627 - 1.1261/ (0.823) | 1.1802/ 0.10060/ 0.02145/ 1.1356/ 1.2248/ **(0.002*)** | 1.1120/ 0.09883/ 0.01003/ 1.0921 - 1.1320/ (0.157) | +0.0917 |

Legend: Mean / Standard Deviation / Standard Error / Mean Lower Bound and Mean Upper Bound (95% Confidence Interval) / p-value of comparisons with group I (p-values highlighted in bold if significant).

**Supplementary Table S2**

**SI-Ratio Comparisons of Group I with Group I, II, IV and all Patient Groups (II–IV) with the Pulvinar Thalami as Reference Value**

|  | **Group I** | **Group II** | **Group III** | **Group IV** | **Group II–IV (all)** | **SI difference Group I vs. Group IV** |
| --- | --- | --- | --- | --- | --- | --- |
| Left caudate nucleus | 0.9445/ 0.058118/ 0.012996/ 0.91730 - 0.97170 | 0.95545/ 0.075011/ 0.016773/ 0.92034 - 0.99056/ (0.563) | 0.93434/ 0.057709/ 0.009755/ 0.91452 - 0.95417/ (0.545) | 0.90179/ 0.046757/ 0.009969/ 0.88106 - 0.92252/ **(0.023*)** | 0.93341/ 0.061613/ 0.006256/ 0.92099 - 0.94582/ (0.356) | −0.04271 |
| Right caudate nucleus | 0.8933/ 0.056872/ 0.012717/ 0.86668 - 0.91992 | 0.89965/ 0.071262/ 0.015935/ 0.8663 - 0.933/ (0.733) | 0.8878/ 0.055769/ 0.009427/ 0.86864 - 0.90696/ (0.739) | 0.85766/ 0.051652/ 0.011012/ 0.83476 - 0.88056/ (0.052) | 0.88454/ 0.059694/ 0.006061/ 0.87251 - 0.89657/ (0.436) | −0.03564 |
| Left putamen | 1.0098/ 0.055863/ 0.012491/ 0.98366 - 1.03594 | 1.01323/ 0.073033/ 0.016331/ 0.97904 - 1.04741/ (0.849) | 0.99779/ 0.052798/ 0.008924/ 0.97965 - 1.01592/ (0.451) | 0.96488/ 0.044897/ 0.009572/ 0.94497 - 0.98478/ **(0.012*)** | 0.99598/ 0.058533/ .005943/ 0.98419 - 1.00778/ (0.215) | −0.04492 |
| Right putamen | 1.0043/ 0.059649/ 0.013338/ 0.97638 - 1.03222 | 0.98855/ 0.076618/ 0.017132/ 0.95269 - 1.02441/ (0.445) | 0.97589/ 0.063942/ 0.010808/ 0.95392 - 0.99785/ (0.122) | 0.94476/ 0.059293/ 0.012641/ 0.91847 - 0.97105/ **(0.04*)** | 0.9773/ 0.067118/ 0.006815/ 0.96377 - 0.99082/ **(0.038*)** | −0.05954 |
| Left globus Pallidus | 1.19435/ 0.050267/ 0.011240/ 1.17082 - 1.21788 | 1.20318/ 0.080401/ 0.017978/ 1.16555 - 1.24080/ (0.675) | 1.1769/ 0.075419/ 0.012748/ 1.15099 - 1.20281/ (0.35) | 1.14368/ 0.046115/ 0.009832/ 1.12324 - 1.16413/ **(0.015*)** | 1.17838/ 0.068681/ 0.006974/ 1.16454 - 1.19222/ (0.241) | −0.05067 |
| Right globus pallidus | 1.1878/ 0.057950/ 0.012958/ 1.16068/ 1.21492 - 1.118 | 1.1729/ 0.085816/ .019189/ 1.13274 - 1.21306/ (0.547) | 1.15793/ 0.093436/ 0.015794/ 1.12583 - 1.19003/ (0.175) | 1.12167/ 0.055180/ .011764/ 1.09720 - 1.14613/ **(0.007*)** | 1.15895/ 0.080082/ 0.008131/ 1.14281 - 1.17509/ (0.063) | −0.06613 |

Legend: Mean / Standard Deviation / Standard Error / Mean Lower Bound and Mean Upper Bound (95% Confidence Interval) / p-value of comparisons with group I (highlighted in bold if significant).

**Supplementary Table S3**

**SI-Ratio Comparisons of Group II with Group IV with Frontal WM as Reference Value (first column) and with the Pulvinar Thalami ( second column)**

|  | **Group II**  **(WM as reference)** | **Group IV**  **(WM as reference)** | **SI Ratio difference**  **Group II vs. IV**  **(WM as reference)** | **Group II**  **(pulv. Thal. as reference)** | **Group IV**  **(pulv. Thal. as reference)** | **SI Ratio difference**  **Group II vs. IV**  **(pulv. Thal. as reference)** |
| --- | --- | --- | --- | --- | --- | --- |
| Left caudate nucleus | 0.88458/ 0.048801/ 0.010912/ 0.86174 - 0.90741 | 0.90524/ 0.051296/ 0.010936/ 0.88250 - 0.92799/ (0.136) | 0.02066 | 0.95545/ 0.075011/ 0.016773/ 0.92034 - 0.99056 | 0.90179/ 0.046757/ 0.009969/ 0.88106 - 0.92252/ **(0.004*)** | -0.05366 |
| Right caudate nucleus | 0.88478/ 0.036872/ 0.008245/ 0.86752 - 0.90203 | 0.90077/ 0.055898/ 0.011917/ 0.87598 - 0.92555/ (0.232) | 0.01599 | 0.89965/ 0.071262/ 0.015935/ 0.8663 - 0.933 | 0.85766/ 0.051652/ 0.011012/ 0.83476 - 0.88056/ **(0.023*)** | -0.03564 |
| Left putamen | 0.93818/ 0.041558/ 0.009293/ 0.91873 - 0.95762 | 0.96874/ 0.051869/ 0.011059/ 0.94574 - 0.99174/ **(0.027*)** | 0.03056 | 1.01323/ 0.073033/ 0.016331/ 0.97904 - 1.04741 | 0.96488/ 0.044897/ 0.009572/ 0.94497 - 0.98478/ **(0.007*)** | -0.04835 |
| Right putamen | 0.9721/ 0.036163/ 0.008086/ 0.95518 - 0.98902 | 0.992/ 0.063563/ 0.013552/ 0.96382 - 1.02018/ (0.20) | 0.03364  (0.20) | 0.98855/ 0.076618/ 0.017132/ 0.95269 - 1.02441 | 0.94476/ 0.059293/ 0.012641/ 0.91847 - 0.97105/ **(0.032*)** | -0.04379 |
| Left globus Pallidus | 1.11558/ 0.058245/ 0.013024/ 1.08832 - 1.14283 | 1.14922/ 0.070869/ 0.015109/ 1.11780 - 1.18064/ (0.084) | 0.03364  (0.084) | 1.20318/ 0.080401/ 0.017978/ 1.16555 - 1.24080 | 1.14368/ 0.046115/ 0.009832/ 1.12324 - 1.16413/ **(0.005*)** | -0.0595 |
| Right globus pallidus | 1.15418/ 0.045931/ 0.010270/ 1.13268 - 1.17567 | 1.17902/ 0.079681/ 0.016988/ 1.14369 - 1.21434/ (0.239) | 0.02484 | 1.1729/ 0.085816/ .019189/ 1.13274 - 1.21306 | 1.12167/ 0.055180/ .011764/ 1.09720 - 1.14613/ **(0.036*)** | -0.05123 |
| Left pulvinar thalamus | 0.93038/ 0.061001/ 0.013640/ 0.90183 - 0.95892 | 1.00648/ 0.072199/ 0.015393/ 0.97447 - 1.03850/ **(<0.001*)** | 0.0761 | - | - | - |
| Right pulvinar thalamus | 0.98868/ 0.059586 0.013324 0.96079 1.01656 | 1.05356/ 0.083831 0.017873 1.01639 1.09073 **(0.004*)** | 0.06488 | - | - | - |
| Left dentate nucleus | 1.0166/ 0.10254/ 0.02293/ 0.9686 - 1.0646 | 1.1210 .08438 .01799 1.0836 1.1584/ **(0.003*)** | 0.1044 | - | - | - |
| Right dentate nucleus | 1.0916/ 0.09262/ 0.02071/ 1.0482 - 1.1349 | 1.1802/ 0.10060/ 0.02145/ 1.1356 1.2248/ **(0.005*)** | 0.0886 | - | - | - |

Legend: Mean / Standard Deviation / Standard Error / Mean Lower Bound and Mean Upper Bound (95% Confidence Interval) / p-value of comparisons between group II and group IV (p-values highlighted in bold if significant).

**Supplementary Table S4**

**SI-Ratio (Dentate Nucleus to Pons and frontal white matter to pons) Comparisons of Group I with Groups II, III, IV and All Patient Groups (II–IV). Comparisons of Group II with Group IV.**

|  | **Group I** | **Group II** | **Group III** | **Group IV** | **Groups II-IV (all)** | **SI difference Group I vs. Group IV** | **SI difference Group II vs Group IV** |
| --- | --- | --- | --- | --- | --- | --- | --- |
| Left dentate nucleus | 1.11781/ 0.069675/ 0.01558/ 1.085204- 1.150421 | 1.0475/ 0.116407/ 0.020907/ 1.004802 - 1.0902/ **(0.014*)** | 1.06121/ 0.099315/ 0.015325/ 1.030264 - 1.092161/ **(0.037*)** | 1.04965/ 0.093950311 0.017152901 1.01458 - 1.084738/ **(0.018*)**/ (0.932) | 1.06414/ 0.10049/ 0.009061/ 1.046204- 1.082078/ **(0.008*)** | -0.06816 | 0.00215 |
| Right dentate nucleus | 1.13087/ 0.064221/ 0.01436/ 1.100809- 1.160921 | 1.06965/ 0.114619/ 0.020586/ 1.027604 - 1.111689/ **(0.035*)** | 1.07704/ 0.104226/ 0.016082/ 1.044557 - 1.109515/ (0.05) | 1.05471/ 0.096909/ 0.017693/ 1.018527 - 1.0909/ **(0.009*)**/ (0.561) | 1.07848/ 0.10181/ 0.00918/ 1.060309- 1.096654/ **(0.010*)** | -0.07616 | -0.01494 |
| Left frontal white matter | 1.0782/ 0.10200/ 0.02281/ 1.0305 - 1.1259 | 1.0470/ 0.12287/ 0.02747/ 0.9895 - 1.1045/ (0.429) | 1.0321/ 0.14249/ 0.02409/ 0.9832 - 1.0811/ (0.189) | 0.9462/ 0.11117/ 0.02370/ 0.8969 - 0.9955/ **(0.001*)/ (0.01*)** | 1.0252/ 0.13066/ 0.01327/ 0.9989 - 1.0515/ **(0.029*)** | -0.132 | -0.1008 |
| Right frontal white matter | 1.0454/ 0.10252/ 0.02292/ 0.9974 - 1.0934 | 0.9972/ 0.11062/ 0.02473/ 0.9454 - 1.049/ (0.234) | 0.9984/ 0.1480/ 0.02502/ 0.9475 - 1.0492/ (0.19) | 0.9088/ 0.12515/ 0.02668/ 0.8533 - 0.9643/ **(0.001*)/ (0.027*)** | 0.9875/ 0.13367/ 0.01357/ 0.9606 - 1.0145/ **(0.027*)** | -0.136 | -0.0884 |

Legend: Mean / Standard Deviation / Standard Error / Mean Lower Bound and Mean Upper Bound (95% Confidence Interval) / p-value of comparisons between group I and other groups / in column 5 (group IV): p-value of comparisons between group II and group IV (p-values highlighted in bold if significant).
